# The Prevalence of Psychological Disorders among Children with Diabetes Aged 5-12 Years Old Referred to the Endocrinology Clinic of Mofid Hospital, Tehran, Iran in 2014-2015

**Published:** 2018

**Authors:** Ghazal ZAHED, Marjan SHAKIBA, Kimia SEIFI

**Affiliations:** 1 Department of Child and Adolescent Psychiatry, Mofid Children Hospital,; 2 Shahid Beheshti University of Medical Science, Tehran, Iran; 3 Department of Endocrinology and Metabolism, Mofid Children Hospital, Shahid Beheshti University of Medical Science, Tehran, Iran; 4 Young Researchers and Elite Club, Roudehen Branch, Islamic Azad University, Roudehen, Iran.

**Keywords:** Psychological disorders, Children, Diabetes

## Abstract

**Objective:**

As there were a few studies on the mental disorders resulting from diabetes in children, this study aimed at investigating the prevalence of psychological disorders among children.

**Materials & Methods:**

We enrolled 323 children with diabetes type 1 aged 5-12 yr old referring to Endocrinology Clinic of Mofid Hospital, Tehran, Iran in 2014-2015. In addition, 317 healthy children were considered as control group. The materials used for data analysis were information form and questionnaire CSI-4 filled out by their parents. The filled questionnaires were rated in that day and then analyzed and diagnosed by the Pediatric Psychiatrist in order to determine the type and intensity of psychological disorder. Results were analyzed using SPSS 20. *T* tests, Scheffe post hoc test and Pearson’s correlation test were used for analysis of data. The amounts were significantly different at *P*<0.05.

**Results:**

In terms of Neuro-Evolutionary disorders, Attention-deficit (ADD), hyperactivity (HD) and Attention-deficit hyperactivity disorders (ADHD) in children with diabetes were significantly higher than those in healthy children (*P*=0.001). Severe fundamental depressive disorders were higher in diabetic children (*P*=0.001). In terms of anxiety disorder, a specific phobia and panic was significantly higher in diabetic children (*P*=0.005). Regarding aggressive behaviors, diabetic children were more disobedient and stubborn than the others.

**Conclusion:**

The prevalence of psychological disorders among diabetic children was higher than that of the others. As psychological disorders will effect on the life quality of children, improvement of life quality of diabetic children and adolescents, on-time diagnosis and treatment of psychological disorders in these patients seems essential.

## Introduction

By development of medical sciences during the past decades, disease patterns in patients have moved towards the chronic diseases and with regard to improving medical cares and increasing the life expectancy, considering the mental and social dimensions of this kind of diseases seems necessary (1). 

Diabetes is one of the most prevalent endocrine and metabolic disorders in children including almost one per 300-500 thousand children under 18 yr old. Diabetes causes pathophysiological changes in whole body, which imposes many problems on the patient and health system (2). In Iran, there are more than 200,000 patients with diabetes type 1 and the prevalence of diabetes is estimated to be 5-7% (3). According to the reports of Iranian Association of Diabetes, about 1.5 million patients with diabetes were recognized in Iran until 1993. Treatment and control of this disease requires heavy costs (4). Diabetic patients are hospitalized 5-10 times more than non-diabetic persons.

Specialized services and emergency in diabetic patients are used 5 times more compared to normal persons. These issues cause diabetes to be one of the health issues (5). The incidence of having diabetes in both preschool and puberty age groups is increasing, though in most communities, there have been changes in occurring and the age of having diabetes in recent decades. Regarding that childhood period is an important period of growth from biological, mental and cognitive aspects and growth and evolution of children depend not only on their biological maturation but also the environmental condition they live and regarding their growth level, children show reaction against adaptation to environmental factors like orphanage, kindergarten, school, hospital and their home, so during childhood, a period of disease means serious damage and the postponement can lead to a serious disorder to growth and evolution of children (6). Regarding the prevalence level of this disease and the needs, patients face with during specific stages of growth and adolescence, WHO declares that, children’s needs must be prioritized and supporting the mental and physical health of children can be accounted as one of the best investments for social and economic development of countries and to this end, children’s health must be fully regarded (7).

The inconvenience that a chronic disease such as diabetes imposes on the person is an issue, and is not recognized from the viewpoint of a physician or nurse. It depends on what the patient feels. The effect of diabetes and its side effects on the life quality have been unknown so far and the studies done on psychological diseases related to diabetes are limited (3). Body and mind are two aspects of human, which effect on one another continually and they can determine one another’s condition (8). Suffering from chronic diseases can effect on different dimensions of life, as Tylor mentions: suffering from chronic diseases effects on patients’ interactions with physical and social environment they live and changes their relations with their peers (9). Physical illnesses can cause mental disorders chemically and physiologically. Patients with chronic physical diseases will have mental disorders and problems (8).

Health definition by WHO is as follows: Health involves in all physical, mental and social aspects, but not simply recovery from illness. Life can be classified into both quantitative (life years from birth to death) and qualitative (the life quality in every period of living years). In a general view, health should not only measure the prevalence and intensity of diseases, but also the estimation of understandings and viewpoint of patients or healthy people about their health is essential. The aim of health care is improving the physical condition and general health of individuals (healthy people and patients) which traditionally depends on diagnosis and treatment of physiological diseases and this method results in disregarding the concepts of health, general efficiency, life quality of individuals and superficial look at them. Of course, there have been changes in this viewpoint and now, the major issue in the assessment of health cares and interventions is information based on the individuals’ comments (Healthy and patient). This assessment aims at providing precise measurement of health in individual and society and evaluating the advantages and disadvantages resulting from health cares (10).

In 1977, a Psychiatrist named Engel, proposed the biopsychosocial pattern for the first time. He believes that biological, psychological and social factors in individuals’ lives effect on their health and illnesses (1). Since then, the investigation of chronic diseases like diabetes has begun with Bio-psycho approach (11). Diabetes provides limitations for diabetic patients which results in decreasing mental health (12). Psychological stress is able to increase blood sugar by activating Hypothalamic-Pituitary-Adrenal axis.

Depression has a mutual relationship with diabetes called a risky factor for having diabetes (13). Most of psychological issues result from problems imposed by diabetes like diet, activity limitation, invasive monitoring blood sugar, daily injection of insulin, chronic physical complications, being hospitalized in hospital and shortening life expectancy (14). A normal emotional response is placed at the beginning of depression, but depression will occur by progression of disease. Defense mechanism of denial and minimization can result in refusing diagnosis and observing treatment. Mental disorders in diabetic patients are more common than the public, nevertheless, most of them remain undiagnosed (15, 16).

Mental disorder in patients with chronic disorders comes along bad quality of life, lack of cooperation with treatment and misusing the medical services. The variables such as gender, obesity, type of treatment, blood sugar increase and complications of diabetes disrupt the life quality of patients independently (14). Diabetic patients have depression background and hypochondriac attitudes (17). Depression, anxiety and adjustment disorders are the most common mental disorders among diabetic patients and is more common in patients with chronic complications (15, 18, 19). Depressed diabetic patients do not follow food and medicine diet so much, they had poorer control of blood sugar, poorer physical and mental function, more physical complications and frequent reference to emergency and hospitalization and spend more on mental health (20). In addition, depression in diabetic patients is prevailed by 44% and the level of depression in diabetic patients who have this kind of disease more than 10 yr is more than the other groups (7). Diabetic patients who lacked emotional disorder had more self -esteem and less depression than the patients with emotional disorder (21). The mean of anxiety, depression, disruptive disorder and shyness in adolescents with diabetes was more than that of adolescents without diabetes and they had more troubles with their family (22).

Regarding that there have been a few studies on the psychological disorders resulting from diabetes in children, this study was done aiming at investigating the mental disorders in children aged 5-12 yr old.

## Materials & Methods

This study was done by case control practice, which investigated the relationship between diabetes, and the prevalence of psychological disorders in diabetic children referring to Mofid Hospital, Tehran, Iran in 2014-15. To this end, the mental health of all children with diabetes aged 5-12 yr old referring to Endocrine Clinic of the hospital (morning and evening) was evaluated and the required services were provided in accordance with problems they had and the duplicate cases were not considered. 

First, the psychologists of hospital informed the parents about the aims of study and after getting informed consent from parents, the information form was filled out. Questionnaire CSI-4 was filled out by parents. The filled questionnaires were rated in that day and then were evaluated by pediatric psychiatrist in order to determine the type and intensity of psychological disorder. All of children who were diagnosed to have psychological disorders, interviewed and evaluated clinically by the psychiatrist. Samples under study included all of children with diabetes 1 aged 5-12 yr old. Sampling was done continuously and the duplicate cases were not considered. The inclusion criteria of diabetic children were the clinical and paraclinical results (BS) and diagnosis recorded in medical records of the clinic and approval of Endocrinologist.

As prevalence of psychological disorders in children aged 5-15 yr without any clinical problem was calculated to be 5.3% (31-32-33), so based on the Cochran formula, the number of samples was 323 (167 girls and 156 boys). Besides, 317 healthy children (children who referred to the clinic of hospital just for growth control or flu and the pediatricians did not record any chronic disease based on their medical history, physical examination and investigation of growth index) were considered as control group (161 girls and 156 boys) and both forms were filled out by their parents. 

**Materials and data collection method:**

The materials of data collection included information form and CSI-4. In information form, variables such as age and gender of children, age of onset of the disease and time duration of having disease, age of child during the time of filling out the form, financial state of family, education level of the child and parents and marital status of parents. CSI-4 questionnaire provides an effective and economical method for monitoring the emotional and behavioral problems aged 5-12 yr old. CSI-4 items are based on the diagnostic criteria determined in Diagnostic and Statistical Manual of Mental Disorders by American Association of Psychiatric. 

There are two versions of CSI-4 for parents and the teacher, which are very similar, and its takes 10 to 15 min to fill it out. CSI-4 needs a lot of time, that is why it is replaced by difficult, time- consuming and expensive interviews in clinical applications and designed based on DSM-IV. The rating is done fast and easily in accordance with rating instruction, criterion scores, diagnosis and classification of symptoms based on T scores. It explains two different ways of counting scores of symptoms based on the symptoms of behavioral and emotional disorders and defines the least score required for diagnosis. The least score is rating criteria. If one child shows the least score of rating for diagnosis of disorder, that child receives “yes” which shows the need for deeper clinical evaluation. The scores of intensity of symptoms measure the deviation degree of a behavior compared to the natural samples. In order to determine the intensity of symptoms, the specified scores are compared to the scores of wide range of children (sample norm). The method of mean (SD) is used for describing the intensity of symptoms. When the scores of symptom intensity is between one and two mean (SD) (for example, T score from 60-69), the intensity of average symptoms will be accepted. 

The score of two or more SDs of mean shows the high intensity of symptoms. Calculating the scores of intensity will be easier by applying the sheet including the scores of symptom intensity. By using this material, hyperactivity disorders with lack of attention, stubbornness disorder, aggressiveness, conduct disorder; anxiety, Schizophrenia, main depression disorder, dysthymic disorder, autism disorder, Asperger disorder, social phobia and Separation Anxiety Disorder are recognized. 

**Statistical Analysis**

Results were analyzed using SPSS 20 (Chicago, IL, USA). Mean (SD) and (%) were used for quantitative and qualitative data, respectively. In order to investigate the difference of mental disorders between the diabetic and normal children, *t* test was used. Scheffe post-hoc test was used to investigate the difference between groups. Pearson correlation was used to determine the relationship between variables. *P *< 0.05 shows significantly difference.

## Results

Comparing the prevalence of psychological disorders in diabetic children and healthy children

Overall, 640 children were investigated, of whom 323 children had diabetes. 51.7% were girls. There were 317 children without diabetes (50.8% girls). In addition, the parents of diabetic children (63.5% fathers and 36.5% mothers) and of healthy children (67.5% fathers and 32.5% mothers) filled the questionnaire. In the group of diabetic children, 13% had favorable, 43.7% average and 43.3% poor financial state and in the group of healthy children, 38.2% had favorable, 65.5% average and 5.4% poor financial state. In order to investigate the mean difference of mental disorders between diabetic and healthy children, *t* test was used. In addition, using the cutting point (the number showing disorder) we could determine the disorder occurrence by comparing the means of each group. 

In terms of Attention-deficit disorder (ADD), in both groups of diabetic and healthy children, there was a significant different between the mean of scores (*P *< 0.001). The mean of scores in diabetic children (96.1%) was higher than that in healthy children (21.1%). The mean of each group was not equal to the cut-off point. Considering hyperactivity disorder (HD), there was a significant difference between the means of both groups (*P *< 0.001). The mean of scores in diabetic children (18.5%), was higher than that in healthy children (58.1%). The mean of each group was not equal to cut-off point. 

The investigation of mean of received score for diagnosis of ADHD in both groups showed that there was a significant difference between the means of both groups (*P *< 0.001). The mean of scores in diabetic children (85.9%), was higher than that of healthy children (35.7%). The mean of each group was not equal to cut-off point. Differences in the other domains (neurodevelopmental and other psychiatric disorders) are shown in Table 1.

**Table 1:** **. **Comparison of means of Neurodevelopmental disorders, depression, anxiety, obsessive-compulsive, elimination, Disruptive behavior disorders in diabetic and healthy children included in items of CSI-4

Variable		Group(No.)	Mean	Cut-off point	SD	*t*	df	*P*
Neuro-developmental disorders	ADD	Diabetic(n=323)	1.96	4	0.100	5.474	638	0.001
		Healthy (n=317)	1.21		0.093			
	HD	Diabetic(n=323)	2.18	6	0.127	3.719	638	0.001
		Healthy (n=317)	1.58		0.099			
	ADHD	Diabetic(n=323)	9.85	12	0.359	4.237	638	0.001
		Healthy (n=317)	2.79		0.145			
	Motor tic	Diabetic(n=323)	0.359	1	0.026	-3.104	638	0.002
		Healthy (n=317)	0.479		0.028			
	vocal tic	Diabetic(n=323)	0.315	1	0.025	-5.778	638	0.000
		Healthy (n=317)	0.536		0.028			
	Autism	Diabetic(n=323)	0.965	6	75.0	-0.222	638	0.824
		Healthy (n=317)	0.993		0.099			
	Asperger	Diabetic(n=323)	0.702	3	0.053	-0.632	638	0.528
		Healthy (n=317)	0.763		0.079			
Depression Disorders	severe Depression	Diabetic(n=323)	4.061	4	0.156	3.482	638	0.001
		Healthy (n=317)	3.384		0.114			
	Dysthymia (poor)	Diabetic(n=323)	2.597	3	0.116	0.117	638	0.907
		Healthy (n=317)	2.580		0.086			
Obsessive-compulsive disorder	Obsession	Diabetic(n=323)	0.390	1	0.027	-2.207	638	0.028
		Healthy (n=317)	0.476		0.028			
	Compulsion	Diabetic(n=323)	0.294	1	0.025	-4.733	638	0.000
		Healthy (n=317)	0.473		0.028			
Anxiety disorders	General Anxiety	Diabetic(n=323)	1.47	3	0.600	-2.391	638	0.017
		Healthy (n=317)	1.76		0.027			
	Specific phobia and panic	Diabetic(n=323)	0.600	4	0.047	2.797	638	0.005
		Healthy (n=317)	0.441		0.035			
	Social phobia	Diabetic(n=323)	0.548	3	0.067	-12.111	368	0.000
		Healthy (n=317)	1.265		0.62			
	Separation disorders	Diabetic(n=323)	0.536	3	0.600	-5.354	368	0.000
		Healthy (n=317)	1.056		0.027			
Elimination disorders	Enuresis	Diabetic(n=323)	0.009	1	0.005	1.721	638	0.086
		Healthy (n=317)	0.000		0.0000			
	Stubbornness and disobedience	Diabetic(n=323)	1.55	4	0.098	8.011	638	<0.001
		Healthy (n=317)	0.630		0.058			
	Conduct Disorder	Diabetic(n=323)	3.74	3	0.177	0.966	638	0.334
		Healthy (n=317)	3.53		0.130			

**assessing the effect of different variables on prevalence of psychological disorders in diabetic children**

Only the attention deficit between diabetic children of both genders (boys and girls) was significantly different (*P *< 0.05), except for disorders like social phobia and autistic symptoms, the mean of psychological disorders in diabetic girls was higher. Based on the Scheffe post hoc test, financial state had a significant effect on occurrence of main depression in diabetic children (*P *= 0.039) and its relationship with other psychological disorders was not significant. 

ADHD predominantly overactive type and main depression disorder (MDD) among diabetic children with different financial states were significantly different at *P *= 0.018 and *P *= 0.039, respectively. In order to determine the differences in the group of financial states (good, average and poor), Scheffe post hoc test was used.

**Table 2:** Scheffe post hoc test for investigating the differences between groups in terms of severe depression disorder

	**Financial state**	**Financial state**	**Mean Difference (I-J)**	**Std. Error**	**Sig.**	**95% Confidence Interval**	
**severe depression **	Good	Average				**Lower Bound**	**Upper Bound**
		Poor	.12158	.49092	.970	-1.0857	1.3289
	Average	Good	-.70476	.49133	.359	-1.9131	.5035
		Poor	-.12158	.49092	.970	-1.3289	1.0857
	Poor	Good	-.82634^*^	.33320	.048	-1.6458	-.0069
		Average	.70476	.49133	.359	-.5035	1.9131
			.82634^*^	.33320	.048	.0069	1.6458

The results from Scheffe post hoc test about severe depression disorder suggested that there was a significant difference between groups with average and poor financial states in terms of this kind of disorder (*P*<0.05) (Table 2). There was a significant difference between diabetic children with average and those with poor financial states in terms of occurrence of symptoms of severe depression disorder. 

**Table 3:** Scheffe post hoc test for investigating the difference between groups in terms of ADHD

	Financial state	Financial state	**Mean Difference (I-J)**	**Std. Error**	**Sig.**	**95% Confidence Interval**	
ADHD	Good	Average	-.66768	1.12622	.554	**Lower Bound**	**Upper Bound**
		Poor	-2.39762^*^	1.12714	.034	-2.8834	1.5480
	Average	Good	.66768	1.12622	.554	-4.6152	-.1801
		Poor	-1.72994^*^	.76438	.024	-1.5480	2.8834
	Poor	Good	2.39762^*^	1.12714	.034	-3.2338	-.2261
		Average	1.72994^*^	.76438	.024	.1801	4.6152
			-.66768	1.12622	.554	.2261	3.2338

The results from Scheffe post hoc test about ADHD suggested that there was a significant difference between groups with good and average, and average and poor financial states in terms of this kind of disorder (*P*<0.05) (Table 3).

There was a significant difference between diabetic children with poor and those with good and average financial states in terms of occurring the symptoms of ADHD.

Based on Pearson correlation among the mental disorders, autism (*P*<0.05) and Asperger (*P*<0.001) disorders had significant relationship with age of diabetic patients (Table 4).

**Table 4:** Investigating the relationship between time duration of disease and psychological disorders of diabetic patients

	**Pearson Correlation**	**Sig. (2-tailed)**
**Attention Deficit (AD)**	-0.019	0.735
**Hyperactivity **	-0.025	0.655
**ADHD**	-0.004	0.938
**Stubbornness **	-0.011	0.841
**Conduct disorder **	0.07	0.207
**Anxiety **	-0.076	0.172
**Phobia **	-0.093	0.094
**Obsession**	0.007	0.898
**Compulsions**	-0.23	0.676
**PTSD**	-0.022	0.692
**Motor tic**	-0.57	0.310
**vocal tic **	0.043	0.438
**Psychosis**	0.040	0.478
**severe depression **	0.054	0.335
**Poor depression**	0.014	0.803
**Autism **	0.090	0.106
**Asperger **	0.076	0.173
**Social phobia**	-0.050	0.372
**Social anxiety **	-0.127*	0.023
**Enuresis**	-0.030	0.587

There was a significant relationship between mental disorders, social anxiety disorder (*P*<0.05) and time duration of disease in diabetic children. 

## Discussion

In this study, psychological disorders resulting from diabetes in children aged 5-12 yr old were investigated. Diabetes is ever increasing disease. Regarding the fact that childhood is an important period of growth in terms of biological, mental and cognitive aspects and during childhood, a period of disease can be a real harm and a delay can cause a serious disorder in growth and evolution of child. Therefore, diagnosis of psychological disorders in diabetic children is an important issue. 

As a whole, the results from this study suggest that there was a significant difference between the mean of scores in both groups (diabetic and non-diabetic children) in terms of mental disorders indexes based on CSI-4 questionnaire (*P*<0.05). In this regard, the mean of scores in diabetic children in terms of AD, ADHD, MDD, ODD, specified phobia and panic was higher than healthy children were. In a study, the level of psychological disorders in diabetic children was three times more than normal children was, and the most common psychological disorders were anxiety and depression (23). This result is consistent with our study to some extent. In Mashad, the most prevalent mental disorders in diabetic patients were physical disorder (89%), depression (87%) and anxiety (78%) (24). Besides, 54% of diabetic patients were suspicious to psychological disorders and further diagnostic investigations were needed (25). In a study, the most prevalent mental disorders among diabetic patients were adjustment disorders (15%), main depression (13.75%) and anxiety (6.25%) (26). In our study, MDD, ADHD, ODD were the most prevalent psychological disorders in diabetic disorders. In Taiwan, there was a significant relationship between ADHD and Diabetes Mellitus type 2 (OR= 2.75, CI: 1.82-4.16) which is consistent with the results our study (27). They believe that diabetes mellitus type 2 effects on frontal lobe and hippocampus and results in ADHD. In the present study, suffering from MDD in diabetic patients was higher and the previous studies done about this issue approve it (28, 29).

The stress resulting from diabetes, poor control of hemoglobin AIC, BMI and negative life events effect on MDD occurrence (30). In the present study, specified phobia and panic in diabetic patients was higher. It was shown that the prevalence of anxiety disorders in patients with diabetes was three times more than the normal population (31). 

Regarding that diabetes is a chronic and disabling disease, it will effect on all areas of life and as a result, psychological problems are prevalent in these patients but regarding that more prevalence of anxiety disorder (generalized anxiety disorder, separation anxiety and social anxiety disorder) and OCD in our study, we can point to ever increasing of prevalence of psychological disorders among children in Iran which is worrying and needs more investigations and research. 

**Limitations**

The attitude and cooperation of parents in filling the questionnaire were affected by the socioeconomic level and education level of patients, which could effect on the reliability of results. Applying the self-report tools is problematic and the testes may not be honest enough to express their problems and answer the questionnaires. These conditions were not controllable for the researches.

**In conclusion**, the prevalence of ADHD (Hyperactivity- Attention Deficit) and MDD (Main depression) in diabetic children is more than healthy children is.

Regarding that one of the most common concerns among diabetic children and adolescents is lack of cooperation in therapy and diet control and as pediatric psychological disorders effect on the issue relating to cooperation of patient, it is necessary to increase the cooperation of the patient with diabetes treatment diets by on time recognition of these disorders. On the other hand, one of the effective factors of improving the quality of life of diabetic children and adolescents is on time recognition and treatment of psychological disorders in these patients and with regard to its considerable prevalence among this group, its importance is obvious.

## References

[1] Hashemian K (1999). [Psychology of health].

[2] Powers A, Kasper D, Fauci A, Longo D (2006). Diabetes Mellitus. Harrison's Endocrinology.

[3] Mortazavi H, Tabatabaii Chehr M (2003). Textbook of pediatric nursing.

[4] Rekabdar M (2000). Appling of distinction analysis for diagnosing of diabetic patients in Tehran [dissertation].

[5] World Health Organization ( 1994). WHO study group prevention of diabetes mellitus.

[6] Bortolote GS, Bretas JR (2008). [The stimulating environment for the development of hospitalized children]. Rev Esc Enferm USP.

[7] Ghasemababdi R, Mohammadkhani P ( 2000). Life skills training courses.

[8] Taylor E (1999). Nursing Children.

[9] Centers for Disease Control Diabetes in Managed Care Work Group (2001). Diabetes mellitus in managed care: complications and resource utilization. Am J Manag Care.

[10] Amini P (2001). Study of problems in children and adolescents with type 1 diabetes in Isfahan metabolism research center. J Nursing Faculty.

[11] Bruner LS, Swarth DI (2008). Liver, Glands and Ducts.

[12] American Diabetes Association (2011). Standards of medical care in diabetes--2011. Diabetes Care.

[13] Sridhar GR (2007). Psychiatric co-morbidity & diabetes. Indian J Med Res.

[14] Koopmanschap M (2002). Coping with Type II diabetes: the patient's perspective. Diabetologia.

[15] Das-Munshi J, Stewart R, Ismail K, Bebbington PE, Jenkins R, Prince MJ (2007). Diabetes, common mental disorders, and disability: findings from the UK National Psychiatric Morbidity Survey. Psychosom Med.

[16] Shafiei K, Amini M (1999). [Psychological problems in diabetic patients]. Res Med Sci.

[17] Livneh H, Wilson LM (2016). Coping Strategies as Predictors and Mediators of Disability-Related Variables and Psychosocial Adaptation. Rehabil Couns Bull.

[18] Kruse J, Schmitz N, Thefeld W, German National Health I, Examination S (2003). On the association between diabetes and mental disorders in a community sample: results from the German National Health Interview and Examination Survey. Diabetes Care.

[19] Winocour PH, Main CJ, Medlicott G, Anderson DC (1990). A psychometric evaluation of adult patients with type 1 (insulin-dependent) diabetes mellitus: prevalence of psychological dysfunction and relationship to demographic variables, metabolic control and complications. Diabetes Res.

[20] Harris MD (2003). Psychosocial aspects of diabetes with an emphasis on depression. Curr Diab Rep.

[21] Hosseini J (1994). Survey of depression rate among diabetic patients with type II of diabetes in the educational therapeutic center of Amol City [dissertation].

[22] Vaghar Anzabi M (1999). Comparison of emotional disorder and self-esteem between diabetic and non-diabetic patients [dissertation].

[23] Blanz BJ, Rensch-Riemann BS, Fritz-Sigmund DI, Schmidt MH (1993). IDDM is a risk factor for adolescent psychiatric disorders. Diabetes Care.

[24] Abdollahian E, Mokhber N (2000). [Study of psychopathologies in non insulin dependent diabetes mellitus patients]. J Mashhad Univ Med Sci.

[25] Larijani B, Bayat MK, Gorgani MK, Bandarian F, Akhondzadeh S, Sadjadi SA (2004). Association between depression and diabetes. German J Psychiatry.

[26] Alireza S, Bakhshani N, Baghban-Haghighi M, Samadi R, Lashkaripoor K, Safarzai M (2012). Prevalence of psychiatric disorders in patients with diabetes type 2. Zahedan J Res Med Sci.

[27] Chen HJ, Lee YJ, Yeh GC, Lin HC (2013). Association of attention-deficit/hyperactivity disorder with diabetes: a population-based study. Pediatr Res.

[28] Golden SH, Lazo M, Carnethon M (2008). Examining a bidirectional association between depressive symptoms and diabetes. JAMA.

[29] Rubin RR, Ciechanowski P, Egede LE, Lin EH, Lustman PJ (2004). Recognizing and treating depression in patients with diabetes. Curr Diab Rep.

[30] Scherrer JF, Garfield LD, Chrusciel T (2011). Increased risk of myocardial infarction in depressed patients with type 2 diabetes. Diabetes Care.

[31] Grigsby AB, Anderson RJ, Freedland KE, Clouse RE, Lustman PJ (2002). Prevalence of anxiety in adults with diabetes: a systematic review. J Psychosom Res.

